# Supplementary material for: Prevalence of abnormal urinary cadmium and risk of albuminuria as a primary bioindicator for kidney problems among a healthy population
Source: PeerJ. 2021 Aug 19;9:e12014. doi: 10.7717/peerj.12014 (PMC8380425; doi:10.7717/peerj.12014)
Supplement: Supplemental Information 2 [file peerj-09-12014-s002.docx]

**BORANG SOAL SELIDIK**

**BAHAGIAN A: SOSIODEMOGRAFI & SOSIOEKONOMI**

1. Jantina?

[ ] Lelaki

[ ] Perempuan

1. Bangsa/Kumpulan Etnik:

[ ] Melayu

[ ] Cina

[ ] India

[ ] Lain-lain (Nyatakan)_______________

1. Agama:

[ ] Islam

[ ] Buddha

[ ] Hindu

[ ] Kristian

[ ] Lain-lain (Nyatakan) ____________________________________________

1. Status perkahwinan:

[ ] Bujang

[ ] Berkahwin

[ ] Bercerai/berpisah

[ ] Balu/duda

1. Status pekerjaan:

[ ] bekerja

[ ] tidak bekerja, sila terus ke soalan nombor 7.

1. Tahap pedidikan tertinggi:

[ ] tiada pendidikan formal

[ ] sekolah rendah

[ ] sekolah menengah

[ ] peringkat kolej dan universiti

1. Pendapatan sebulan: RM__________
2. Purata pendapatan isi rumah: RM __________
3. Jumlah isi rumah: ___ orang
4. Sudah berapa tahunkah anda telah bermastautin di Kepong?

______tahun

**BAHAGIAN B: STATUS KESIHATAN**

1. Adakah anda mengalami masalah kesihatan berikut?

Kanser [ ]

Kencing manis [ ]

Darah tinggi [ ]

Penyakit buah pinggang [ ]

Penyakit jantung [ ]

Penyakit lain (Nyatakan)___________________

1. Adakah anda sedang mengambil sebarang ubatan?

Ya [ ]

Tidak [ ]

Jika ya, sila nyatakan jenis ubat dan tempoh pengambilan

__________________________________________________________________________________________________

Soalan 13 dan 14 perlu ditanya kepada peserta wanita sahaja.

1. Adakah puan sedang hamil?

Ya [ ]

Sudah berapa bulankah kandungan puan? _____bulan

Tidak [ ]

1. Waktu terakhir datang haid? _______________________
2. Adakah anda terlibat dengan apa-apa aktiviti fizikal atau senaman dalam masa 48 jam yang lalu?

Ya [ ] Sila nyatakan aktiviti dan tempoh masa melakukannya__________

Tidak [ ]

1. Adakah anda merokok?

Ya [ ] Sila jawab soalan 17.

Tidak [ ] Abaikan soalan 17.

1. Jumlah rokok yang telah dihisap sehingga sekarang?

Kurang daripada 100 batang [ ]

100 batang dan ke atas [ ]

**BAHAGIAN C: PEMERIKSAAN KESIHATAN**

1. Berat

_____kg

1. Tinggi

_____m

1. Indeks Jisim Badan

_____

1. Bacaan tekanan darah

_____mmHg

1. Bacaan denyutan nadi

_____bpm

1. Bacaan gula dalam darah

_____mmol/L

1. Bacaan keputusan urinalisis

__________

1. Bacaan urin kadmium

__________
