# Supplementary material for: Prevalence of abnormal urinary cadmium and risk of albuminuria as a primary bioindicator for kidney problems among a healthy population
Source: PeerJ. 2021 Aug 19;9:e12014. doi: 10.7717/peerj.12014 (PMC8380425; doi:10.7717/peerj.12014)
Supplement: Supplemental Information 3 [file peerj-09-12014-s003.docx]

**Questionnaire**

**PART A: SOCIODEMOGRAPHIC & SOCIECONOMIC INFORMATION**

1. Gender?

[ ] Male

[ ] Female

1. Ethnicity:

[ ] Malay

[ ] Chinese

[ ] Indian

[ ] Others (Please state)_______________

1. Religion:

[ ] Muslim

[ ] Buddha

[ ] Hindu

[ ] Christian

[ ] Others (Please state) ____________________________________________

1. Marital status:

[ ] Single

[ ] Married

[ ] Divorce

[ ] Widow/widower

1. Working status:

[ ] Working

[ ] Not working

1. Highest education level:

[ ] no formal education

[ ] primary

[ ] secondary

[ ] tertiary

1. Monthly income: RM__________
2. Monthly household income: RM __________
3. Number of household: ___
4. Duration of staying in Kepong? ______years

**PART B: HEALTH STATUS**

1. Have you been diagnosed with any of the following conditions?

Cancer [ ]

Diabetes [ ]

Hypertension [ ]

Kidney disease [ ]

Heart disease [ ]

Others (Please state)___________________

1. Are you taking any kind of medication at the moment?

Yes [ ]

No [ ]

If yes, please state the name of medication and duration of taking it.

__________________________________________________________________________________________________

**Question 13 and 14 only applicable for female.**

1. Are you pregnant?

Yes [ ]

Age of pregnancy? _____months

No [ ]

1. When was your first day of last period? _______________________
2. Are you involved with any kind of physical activity for the past 48 hours?

Yes [ ] Please state type of activity and duration__________

No [ ]

1. Do you smoke?

Yes [. ]

No [. ]

1. How many cigarettes have u smoked so far?

Less than 100 [ ]

100 and more [ ]

**PART C: HEALTH SCREENING**

1. Weight

_____kg

1. Height

_____m

1. Body Mass Index

_____

1. Blood pressure

_____mmHg

1. Pulse rate

_____bpm

1. Random blood sugar

_____mmol/L

1. Urinalysis result

__________

1. Urine cadmium result

__________
